# Supplementary material for: Dynalign II: common secondary structure prediction for RNA homologs with domain insertions
Source: Nucleic Acids Res. 2014 Nov 21;42(22):13939–48. doi: 10.1093/nar/gku1172 (PMC4267632; doi:10.1093/nar/gku1172)
Supplement: SUPPLEMENTARY DATA [file supp_gku1172_nar-02021-z-2014-File012.zip › manual/Text/NAPSS.html]

RNAstructure Command Line Help: NAPSS


|  |  |  |
| --- | --- | --- |
|  | RNAstructure Command Line Help NAPSS | - Contents - Index |
| NAPSS is used to improve the prediction of an RNA secondary structure by integrating NMR data. Note that on Macintosh OS X, and some versions of Linux, there is a small default stack limit. To run NAPSS, the stack limit needs to be increased. On the default shell, bash, use *ulimit -s 8912* or higher, depending on your system configuration. If you are using tcsh, use *limit stack 4096* or higher, depending on your system configuration. The limits can be increased as necessary if the given values above are not sufficient. USAGE: NAPSS <seq file> <NMR file> <ct file> [options] Required parameters: <seq file> \| The name of a sequence file containing input data. ??Note that lowercase nucleotides are forced single-stranded in structure prediction.??; <NMR file> \| The name of an NMR file with constraings.; <ct file> \| The name of a CT file to which output will be written. Options which don't require added values: -h, -H, --help \| Display the usage details message.; -pf, -PF, --pseudoknotFree \| Specify that the sequence is DNA, and DNA parameters are to be used. Default is to use RNA parameters.; -v, -V, --version \| Display version and copyright information for this interface. Options which require added values: -c, -C, --constraint \| Specify a folding constraints file to be applied. For Fold, current supported constraints are force pairs, force a nucleotide to be single stranded, and force a nucleotide to be double stranded. Default is to have no constraints applied.; -d, -D, --DotPercent \| Specify a maximum percent energy difference to consider in the dotplot. Default is 5 percent.; -m, -M, --maximum \| Specify a maximum number of structures per matched constraint set. Default is 100 structures.; -p, -P, --percent \| Specify a maximum percent energy difference. For example, 20 would indicate 20%. Default is 0 which means that all structures are outputted.; -pp, -PP, --posPaired \| Specify the name of the positions paired style output file. Default is to have no file specified.; -sh, -SH, --SHAPE \| Specify a SHAPE data file to be used to generate restraints. These restraints specificially use SHAPE pseudoenergy restraints. Default is no SHAPE data file specified.; -si, -SI, --SHAPEintercept \| Specify an intercept used with SHAPE restraints. Default is -0.6 kcal/mol.; -sm, -SM, --SHAPEslope \| Specify a slope used with SHAPE restraints. Default is 1.8 kcal/mol.; -w, -W, --window \| Specify a window size. Default is 0 nucleotides. References: 1. Reuter, J.S. and Mathews, D.H. "RNAstructure: software for RNA secondary structure prediction and analysis." *BMC Bioinformatics*, 11:129. (2010). 2. Hart, J.M., Kennedy, S.D., Mathews, D.H. and Turner, D.H. "NMR-assisted prediction of RNA secondary structure: identification of a probable pseudoknot in the coding region of an R2 retrotransposon." *J. Am. Chem. Soc.*, 130:10233-10239. (2008). | | |
| Visit The Mathews Lab RNAstructure Page for updates and latest information. | | |
